# Serum levels of galectin-1, galectin-3, and galectin-9 are associated with large artery atherosclerotic stroke

**DOI:** 10.1038/srep40994

**Published:** 2017-01-23

**Authors:** Xin-Wei He, Wei-Ling Li, Cai Li, Peng Liu, Yu-Guang Shen, Min Zhu, Xiao-Ping Jin

**Affiliations:** 1 Department of Neurology, Taizhou Hospital, Wenzhou Medical University, Linhai, 317000, China; 2 Medical Research Center, Taizhou Hospital, Wenzhou Medical University, Linhai, 317000, China

## Abstract

The aim of this study was to assess the expression patterns of serum galectin-1 (Gal-1), galectin-3 (Gal-3), galectin-9 (Gal-9), and galectin-3 binding protein (Gal-3BP) and their associations with stroke outcome in large artery atherosclerotic (LAA) stroke. The serum levels of Gal-1, Gal-3, Gal-9, and Gal-3BP were measured by ELISA in 130 patients with LAA stroke and 130 age- and sex-matched controls. Serum samples were collected from the patients on day 1, day 6, and in the 4th week after ischaemic stroke (IS). An unfavourable outcome was defined as a modified Rankin Scale score of >2 on day 90 after IS. Our results indicated that the Gal-3 and Gal-9 levels were higher in patients with LAA stroke than in controls. A higher Gal-3 level was independently associated with an unfavourable outcome both on day 1 and day 6 after IS. In addition, Gal-9 and Gal-1 levels were upregulated on day 6 and in the 4th week after IS, respectively. For Gal-3BP, no difference was detected between patients and controls and no predictive value was found in patients. In conclusion, these findings suggest that the serum levels of Gal-1, Gal-3, and Gal-9 may be associated with LAA stroke.

Galectins (Gals) are a family of β-galactoside-specific lectins that includes at least 15 members that have a conserved carbohydrate recognition domain sequence with different affinities and specificities for β-galactoside^1^. Gals are expressed intracellularly and extracellularly in different cells and are involved in cell adhesion, migration, activation, proliferation, and apoptosis^1^,^2^. Several studies have demonstrated the roles of different members of this family in modulating pathological processes such as inflammation, immunization, and tumour biology^3^,^4^,^5^,^6^,^7^,^8^.

Galectin-1 (Gal-1), galectin-3 (Gal-3), and galectin-9 (Gal-9) are widely studied members of the Gals family. Despite having significant sequence homologies, experimental studies have demonstrated that different Gals may exert diverse and even opposite effects on atherosclerosis. For example, Gal-1 promotes the development of atherosclerosis^9^; Gal-3 exerts either a facilitating or inhibitory influence on atherosclerosis^10^,^11^; and Gal-9 participates in the regulation of atherosclerosis^12^. In addition, some clinical studies have shown that patients with coronary artery disease (CAD) have higher Gal-3 levels^13^ and lower Gal-9 levels in the circulation than healthy controls^14^.

Galectin-3 binding protein (Gal-3BP), which is a member of the macrophage scavenger receptor cysteine-rich domain superfamily of molecules, is a ubiquitously expressed secreted protein^15^,^16^. It binds to ligands such as Gal-3^17^ and Gal-1^18^. Recent studies demonstrated that Gal-3BP is expressed in coronary artery plaques^19^, but its plasma levels do not reflect coronary plaque instability in patients with CAD^20^. In addition, another study reported that Gal-3BP levels are associated with carotid artery lesions^19^.

These controversial conclusions might result from heterogeneity among different populations and the diverse clinical conditions in the available studies. Nevertheless, these data implicate a common theme, which is that Gal-1, Gal-3, Gal-9 and Gal-3BP may play indispensable roles in atherosclerosis and may act as biomarkers for the risk and/or prognosis of atherosclerosis-related diseases. Furthermore, studies concerning the circulating levels of these biomarkers in patients with ischaemic stroke (IS) are limited.

On the other hand, evidence from experimental studies implicates different roles for the Gals family members in the progression of brain damage after IS. For example, Gal-1 is increased after IS and is implicated in neuroprotection against ischaemia^21^,^22^; Gal-3 is actively released under ischaemia/stroke conditions and amplifies and prolongs the inflammatory response^7^; and the expression of Gal-9 is increased after IS and is involved in neuronal injury^23^. However, these studies were performed primarily in animal models or *in vitro*, and few studies have investigated whether these biomarkers in circulation are associated with the stroke severity and/or outcomes in human after IS.

Therefore, the aim of this study was to evaluate the serum levels of Gal-1, Gal-3, Gal-9, and Gal-3BP in patients with large artery atherosclerotic (LAA) stroke and in age- and sex-matched controls, and to determine the long-term changes in the levels of these biomarkers on day 6 and in the 4th week after IS. We also investigated whether these biomarkers were independently associated with stroke severity and functional outcomes at 90 days after IS.

## Results

### Baseline characteristics of the study participants

The study included 130 patients with LAA stroke and 130 age- and sex-matched non-stroke controls. All the participants are of the Han nationality. The basic characteristics of the study subjects are shown in Table 1. Compared with the controls, the LAA patients had higher systolic blood pressure (SBP), fasting blood glucose (FBG), and high-sensitivity C-reactive protein (hs-CPR) levels and included a greater percentage of smokers.

### Correlations between the serum Gal-1, Gal-3, Gal-9, and Gal-3BP levels

In both the control and stroke groups, the serum levels of Gal-9 were significantly correlated with the Gal-1 (controls, r = 0.324, *p* < 0.001; patients, r = 0.339, *p* < 0.001) and Gal-3 levels (controls, r = 0.382, *p* < 0.001; patients, r = 0.215, *p* = 0.014), and the Gal-3 levels were correlated with the Gal-3BP levels (controls, r = 0.181, *p* = 0.040; patients, r = 0.180, *p* = 0.041). In addition, the Gal-1 levels were correlated with the Gal-3 levels in the stroke group (r = 0.178, *p* = 0.043). No other correlations were found within Gal-related or Gal-3BP-related parameters (Supplementary Table S1).

### Higher serum levels of Gal-3 and Gal-9 were associated with LAA stroke

**Figure 1:**
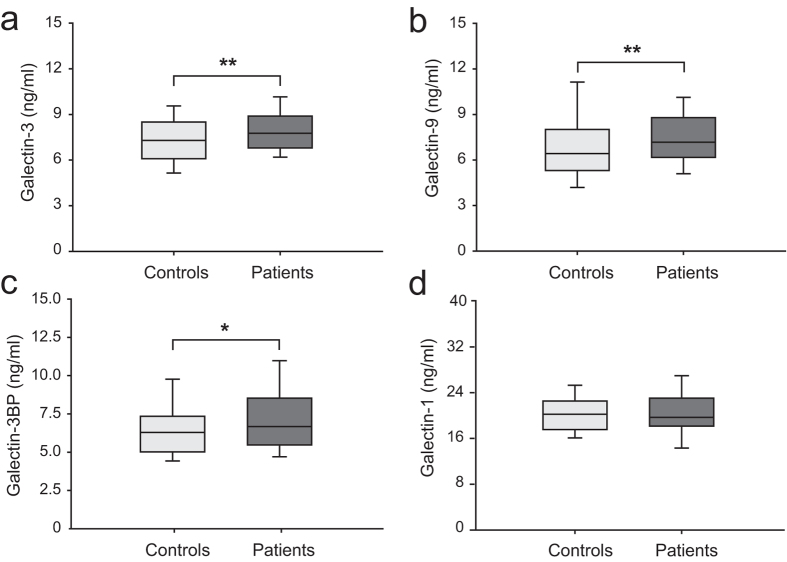
Comparison of the serum levels of galectin-1, -3, -9, and -3BP in the patients and controls. In the box-and-whisker plots, the lower and upper ends of the box represent the 25th and 75th percentiles, respectively; the horizontal line inside the box represents the median value; and the peripheral lines extending to the outer fences represent the 10th and 90th percentiles. Differences between the two groups were compared using Student’s *t*-test (**a,b,d**) or the Mann-Whitney *U* test (**c**). *p < 0.05, **p < 0.01. Abbreviations: galectin-3BP, galectin-3 binding protein.

**Figure 2:**
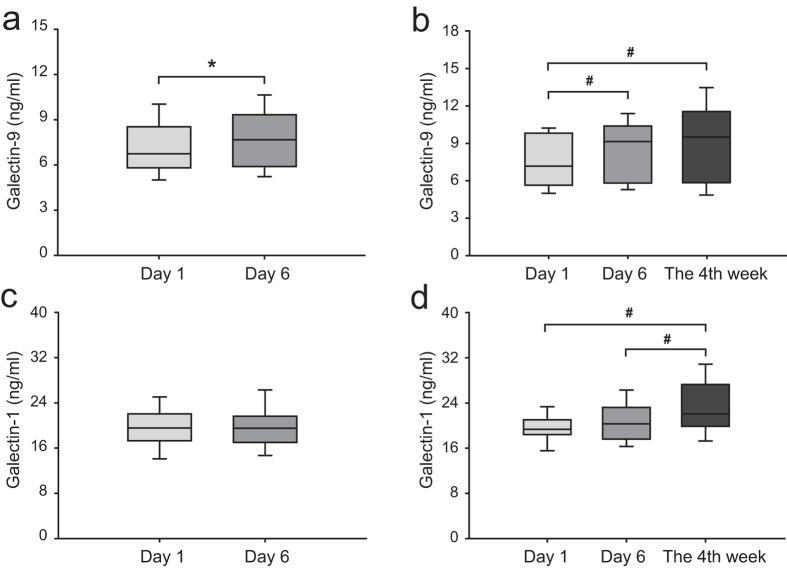
Serum levels of galectin-9 and galectin-1 in patients at different time points after ischaemic stroke. (**a** and **c**) comparisons of galectin-9 and galectin-1 levels between day 1 and day 6 after stroke in 109 patients. The differences were compared using the Wilcoxon signed-rank test (**a**) or a matched *t*-test (**c**). *p < 0.05. (**b** and **d**), comparison of galectin-9 and galectin-1 levels between day 1, day 6, and 4 weeks after stroke in 39 patients. The differences between the three time points were compared using the Friedman *M* test with *post hoc* Wilcoxon signed-rank tests (**b**) and repeated measures ANOVA with *post hoc* matched *t*-tests (**d**). ^#^*p* < 0.0125 was considered statistically significant after the Bonferroni correction.

The serum levels of Gal-3, Gal-9, and Gal-3BP were significantly higher in the patients than in the controls (Figs 1a and 2b, c; Table 2). After adjusting for age, sex, body mass index, hypertension, diabetes, dyslipidaemia, smoking, drinking, homocysteine, creatinine, and hs-CPR, both Gal-3 and Gal-9 remained independent markers of LAA stroke, whereas Gal-3BP did not (Table 2). No difference was found in the Gal-1 levels between the patients and the controls (Fig. 1d).

No correlations were found between the Gal-1, Gal-3, Gal-9, or Gal-3BP levels and stroke severity, which was defined by the stroke volumes and the National Institutes of Health Stroke Scale (NIHSS) scores in the patients (Supplementary Table S2).

### A higher serum level of Gal-3 was associated with an unfavourable outcome on day 90 after IS

Of the Gals and Gal-3BP parameters, the serum levels of Gal-3 were significantly higher in the patients with an unfavourable outcome than in the patients with a favourable outcome, according to the univariate analysis (Table 3; day 1, 7.48 ± 1.64 ng/ml vs. 8.25 ± 1.66 ng/ml, *p* = 0.013; day 6, 7.64 ± 1.80 ng/ml vs. 8.52 ± 2.03 ng/ml, *p* = 0.024), and the clinical variables associated with an unfavourable outcome were older age and higher NIHSS scores (Table 3). After adjusting for these clinical variables, a higher Gal-3 level independently predicted an unfavourable outcome on day 90 after IS (day 1, adjusted odds ratio [95% confidence interval], 1.966 [1.136, 3.401], *p* = 0.016; day 6, 1.946 [1.042, 3.636], *p* = 0.037). No association was found between the stroke outcomes and the Gal-1, Gal-3BP, and Gal-9 levels (Table 3).

A total of 12 patients (10.3%) died within the 90 day period after IS. Due to the relatively low mortality, the overall ability to distinguish the survivors from the non-survivors in the present study could not be estimated.

### Serum levels of Gal-9 and Gal-1 were upregulated on day 6 and in the 4th week after IS

The time-course analysis showed that the serum Gal-9 levels were higher on day 6 after IS than on day 1 (in 109 patients; Fig. 2a, *p* = 0.016) and remained at higher levels in the 4th week after IS (in 39 patients; Fig. 2b, 4 weeks vs. day 6, *p* = 0.040). There was no difference in the Gal-1 levels between day 1 and day 6 after IS (Fig. 2c, *p* = 0.181); however, the Gal-1 levels were higher in the 4th week after IS (Fig. 2d, 4 weeks vs. day 6, *p* = 0.001). The serum levels of Gal-3 and Gal-3BP remained stable throughout day 1, day 6, and 4th week after IS.

## Discussion

The major findings of this study are as follows: (1) the serum levels of Gal-3 and Gal-9 were significantly higher in the patients with LAA stroke than in the controls; (2) the higher Gal-3 level independently predicted an unfavourable outcome on day 90 after IS; and (3) the serum levels of Gal-1 and Gal-9 were upregulated after IS.

Given that Gal-1, Gal-3, Gal-9, and Gal-3BP may play different roles in atherosclerosis^9^,^11^,^12^,^19^, which is a major cause of cardiovascular disease (CVD), and that the levels of some of these parameters in CVD patients have been determined to be different from healthy controls and/or are associated with surrogate markers of atherosclerosis^13^,^19^,^24^, we postulated that the concentrations of Gal-1, Gal-3, Gal-9, and Gal-3BP might differentiate LAA stroke patients from healthy controls and might as predictors for stroke severity and/or outcomes; however, no published reports have previously assessed these levels in LAA stroke patients. In the present study, we demonstrated that patients with LAA stroke have higher Gal-3 levels than controls. Thrombosis or embolism secondary to atherosclerosis of large arteries is the most important underlying pathology of LAA stroke^25^. The role of Gal-3 in atherosclerosis has been highlighted recently. Some experimental studies have demonstrated the pro-atherosclerotic effects of Gal-3, such as attracting monocytes and macrophages^26^, enhancing macrophage phagocytosis, accelerating macrophage transformation into foam cells^11^,^27^, and inducing the proliferation of vascular smooth muscle cells^28^. In addition, a clinical study suggested that Gal-3 levels are increased in patients with carotid atherosclerosis^29^, who are significantly predisposed to experience cerebral ischaemic events^30^. Additional studies have demonstrated that Gal-3 levels are significantly higher in patients with CAD and are correlated with the severity of CAD^13^,^24^. Together with the present study, these findings may support the assumption that Gal-3 is linked to atherosclerosis. Additionally, we also discovered that patients with LAA stroke have higher Gal-9 levels than controls. In general, Gal-9 appears to play a role in immunity and inflammatory processes, particularly in regulatory T cell development and homeostasis^31^. Regulatory T cells mediate the immune responses that participate in the development of atherosclerosis^32^,^33^.

It should be noted that these data should be interpreted cautiously given that these measurements were obtained after the stroke event had occurred. Indeed, the Gal-9 levels were increased on day 6, and thus it is impossible to determine whether the Gal-9 levels had already changed before the measurements were acquired. The Gal-3 levels remained stable after IS in our study. In addition, a recent prospective study reported that higher Gal-3 levels are potentially a predictive marker for stroke in female patients who have undergone carotid endarterectomy^34^.

On the other hand, given that the expression of Gal-1, Gal-3, and Gal-9 are unregulated in the brain after IS and exert either neuroprotective or neurotoxic functions^7^,^21^,^22^,^23^,^35^,^36^, we postulated that these biomarkers in the circulation might be potential predictors for stroke outcomes; however, these associations have not been previously confirmed in stroke patients. In the current study, we found that a higher level of only Gal-3 was independently associated with an unfavourable outcome on day 90 after IS. Several studies have demonstrated that the level of Gal-3 is an independent predictor of cardiovascular events and/or death in patients with heart failure^37^,^38^, CAD^39^,^40^, and peripheral artery disease^29^. Furthermore, Gal-3 can also predict all-cause and/or cardiovascular-related death and the risk of new-onset heart failure in healthy subjects^13^,^41^,^42^. Our findings reveal the adverse prognostic role of higher Gal-3 levels in patients with IS. The possible function and role of Gal-3 in the central nervous system has not been completely elucidated. The prevailing hypothesis in the literature is that Gal3-dependent TLR4 activation after IS could contribute to enhanced microglia activity, which would expand and prolong the inflammatory response in the brain^7^ and the post-ischaemic inflammatory response that is closely related to the adverse outcomes of stroke^43^.

Our study failed to show a significant association between Gal-1 or Gal-9 levels and short-term stroke outcomes in patients with IS; however, both the Gal-1 and Gal-9 levels were upregulated after IS and were significantly correlated with the Gal-3 levels. Several previous studies have shown that the expression of Gal-1 and Gal-9 are significant increased and exert differential functions in the brain after ischaemic brain injury. Gal-1 plays a role in facilitating long-term neuroprotection and improving functional recovery^21^,^22^,^35^,^36^ and Gal-9 is involved in the inflammatory response and neuronal injury^23^. In addition, previous evidence has shown that brain ischaemic injury stimulates angiogenesis for the formation of new brain microvessels^44^,^45^, which become evident within 2 weeks after IS in animal models^46^. The pro-angiogenesis and pro-arteriogenesis effects of the Gal family (including Gal-1 and Gal-9) have been widely studied^47^. Taken together, all these data implicate roles of the increased levels of Gal-1 and Gal-9 in stroke rather than simple acute behaviors after IS, although these specific prognostic values still need to be clarified, perhaps in studies investigating long-term functional outcomes or other aspects of post-stroke prognosis, such as cognition. Furthermore, cerebral ischaemia can trigger biological reactions in both the brain and systemic circulation. It is unclear whether the increased levels of Gal-1 and Gal-9 are predominantly from the brain due to the breakdown of the blood-brain barrier or from any other tissues, or from overlap during recovery.

There are currently no data reported available regarding Gal-3BP in IS. The present study expands the knowledge of the role of Gal-3BP in patients with LAA stroke. We did not find any differences in the Gal-3BP levels between the patients and controls or that Gal-3BP was related to stroke severity or outcomes in patients. This result is consistent with a previous study in patients with cardiovascular disease that found Gal-3BP levels did not differ between patients with or without CAD^20^.

This study has several limitations. First, although our results were significant, our sample size was relatively small. Further studies utilizing a larger sample size are necessary to confirm our findings. Second, we did not assess the differences based on the location of the atherosclerosis. The greater prevalence of intracranial atherosclerosis rather than extracranial atherosclerosis in Asian populations^48^ may limit the extrapolation of our findings to other populations. Third, because the earliest serum samples were obtained on day 1 after the IS had already occurred, we cannot determine whether the measurements obtained at this time point reflect pre-stroke levels or changes that occurred within the first 24 h. A longitudinal study including serial measurements of serum levels (particularly within the first 24 h after IS) may better identify certain changes that occur secondary to IS. Fourth, the patients who had three blood samples had relatively mild symptoms (lower NIHSS and the modified Rankin Scale (mRS) scores) than the whole patients, which might result in bias of the results. Finally, due to the clinical relevance of our study, we did not completely elucidate the pathophysiological mechanism underlying these findings. Nevertheless, this study constitutes the first evaluation of serum levels of Gal-1, Gal-3, Gal-9, and Gal-3BP in humans with LAA stroke.

In conclusion, our study provides preliminary evidence that the serum levels of Gal-3 are higher in patients with LAA stroke and a higher Gal-3 level might be an independent prognostic marker for IS in this population. These findings are consistent with the hypothesis that the Gal-3 might be used as a non-traditional risk factor for atherosclerosis and might participate in the pathological processes after LAA stroke, which suggests that therapies designed to suppress Gal-3 might have a role in the prevention and treatment of CVD. In addition, our study showed that the Gal-1 and Gal-9 levels are upregulated after IS; however, neither Gal-1 nor Gal-9 are associated with the short-term prognosis of stroke. Further studies are needed not only to provide the exact effect and potential biological mechanisms of Gals in the pathogenesis of atherosclerosis and IS, but also to provide deeper insight into the sources and effects of increased levels of these Gals after IS to determine if they are protective, detrimental or neutral.

## Methods

### Study subjects

The present study was performed in accordance with the ethical guidelines of the 1975 Declaration of Helsinki and was approved by the Medical Ethics Committee of Taizhou Hospital. All participants or their relatives provided written informed consent.

A total of 130 patients with LAA stroke were consecutively recruited from the Department of Neurology of Taizhou Hospital, Zhejiang, China, from December 2014 to August 2015.

In this study, acute IS was diagnosed according to the World Health Organization criteria^49^ combined with the confirmation from brain computed tomography or magnetic resonance imaging. LAA stroke was defined according to TOAST (Trial of Org 10172 in Acute Stroke Treatment) criteria^25^. At least two neurologists reviewed the clinical features and diagnostic test results before assigning the subtype classifications.

Patients were included if they met the following criteria: first-time IS, admission within 24 h after symptom onset, and absence of thrombolysis or interventional therapy. In addition, the following exclusion criteria were applied: any history of stroke, severe coronary heart disease or arrhythmia, a history of tumours, severe hepatic or renal insufficiency, haematological disease, autoimmune disease, chronic inflammation or recent infection, or recent surgery.

Controls consisted of 130 age-matched (blocks of 5 years) and sex-matched healthy individuals who were recruited during physical check-ups and were free of the conditions stated in the exclusion criteria.

### Determination of the severity and functional outcome of acute IS

Stroke severity was assessed based on infarct volume and the National Institutes of Health Stroke Scale (NIHSS; scores range from 0 to 42, with higher scores indicating greater deficits)^50^. We calculated the NIHSS scores on day 1 after IS for all patients and on day 6 after IS for the 109 patients who had second blood samples available on day 6. Stroke volume was evaluated based on initial diffusion-weighted imaging lesion volume (in 118 patients) by an experienced neuroradiologist who was unaware of the clinical and laboratory results, and it was calculated as the sum of the infarction areas on each slice multiplied by the slice thickness.

Using the mRS, functional outcome was assessed on day 90 after IS by a neurologist who was blinded to the clinical presentations and biochemical results of patients. The mRS scores of ≤2 and >2 corresponded to favourable and unfavourable outcomes, respectively.

### Sample collection and measurement

**Figure 3:**
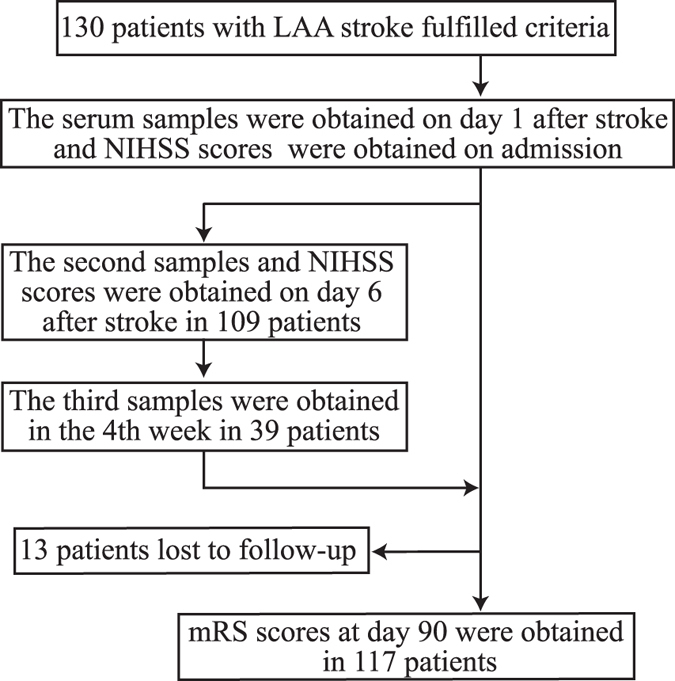
Study flow chart Abbreviations. LAA stroke, large artery atherosclerotic stroke; NIHSS, National Institutes of Health Stroke Scale; mRS, modified Rankin Scale.

A flow chart of the study is shown in Fig. 3. Blood samples were collected in the morning after an overnight fast from 130 patients on day 1 after IS and were also collected from 109 patients on day 6 after IS. In the group of 130 patients, 4 patients died and 17 patients had been discharged or transferred within the first six days following admission. The characteristics of the latter subjects did not significantly differ from those of the 130 patients (Supplementary Table S3). Furthermore, the third blood samples from 39 patients were obtained in the 4th week after IS during their follow-up reviews. These 39 patients had lower NIHSS and mRS scores than those of the 109 patients (Supplementary Table S4). Blood samples were drawn from the control subjects during their physical check-ups after overnight fast.

Measurements of biochemical parameters, such as FBG, triglycerides, and total cholesterol (TC), were performed at the clinical laboratory in the hospital.

Serum samples were stored at −80 °C before being analysed. All samples were thawed only once prior to use. Gal-1, Gal-3, Gal-9, and Gal-3BP levels were measured using commercially available enzyme-linked immunosorbent assay (ELISA) kits (R&D Systems, Minneapolis, MN, USA; Catalogue number DGAL10, DGAL30, DGAL90, and DGBP30B). According to the instructions, the intra-assay and inter-assay coefficients of variation are, respectively, 5.7–8.8% and 7.5–9.5% for Gal-1, 3.0–4.4% and 6.8–8.6% for Gal-3, 3.4–3.7% and 5.2–5.6.0% for Gal-9, and 2.7–3.6% and 4.8–10.0% for Gal-3BP. The mean minimum detectable concentrations of Gal-1, Gal-3, Gal-9, and Gal-3BP are 22 pg/ml, 16 pg/ml, 8 pg/ml, and 22 pg/ml, respectively.

### Data collection and definition

Baseline information was obtained at admission, including race, age, sex, history of health and medication use, smoking status, and alcohol use.

BMI was calculated as measured weight (kg) divided by the square of measured height (m^2^). The risk factors were defined as follows: hypertension (SBP/average diastolic blood pressure (DBP) ≥140/90 mmHg based on the average of two measurements, a history of hypertension, or use of antihypertensive medications), diabetes mellitus (FBG level ≥7 mmol/L, a history of diabetes, or use of antidiabetic treatments), dyslipidaemia (serum triglycerides ≥1.69 mmol/L, low-density lipoprotein cholesterol ≥3.37 mmol/L, high-density lipoprotein cholesterol ≤0.91 mmol/L, or the use of lipid-lowering drugs), smokers (who were currently smoking or had quit smoking within the past year), and alcohol consumers (who consumed ≥2 standard alcoholic beverages per day).

### Statistical analysis

Continuous variables with a normal distribution are expressed as the means ± standard deviations, variables with a skewed distribution are expressed as medians (interquartile range), and variables with a skewed distribution normalized through a log_10_ transformation were expressed as the geometric means ± approximate SD. Categorical data are presented as counts and proportions. For univariate analysis, differences in the clinical data were analysed by Student’s *t*-test, the Mann-Whitney *U* test, and Pearson’s chi-square statistic, as appropriate. The time-course data between two time points were analysed by a matched *t*-test or Wilcoxon signed-rank test, and data between three time points were compared by a repeated measurement ANOVA with *post hoc* matched *t*-tests or the Friedman *M* test with *post hoc* Wilcoxon signed-rank tests, using *the* Bonferroni correction for *p*. Correlation analyses were assessed using either Pearson’s correlation or Spearman’s rank correlation, as appropriate. Multivariate analysis was performed using binary logistic regression analysis adjusted for potential influencing factors. Data analyses were performed using SPSS version 18.0 (SPSS Inc., Chicago, IL, USA). *p* < 0.05 was considered statistically significant unless otherwise noted.

## Additional Information

**How to cite this article**: He, X.-W. *et al*. Serum levels of galectin-1, galectin-3, and galectin-9 are associated with large artery atherosclerotic stroke. *Sci. Rep.* **7**, 40994; doi: 10.1038/srep40994 (2017).

**Publisher's note:** Springer Nature remains neutral with regard to jurisdictional claims in published maps and institutional affiliations.

## Supplementary Material

Supplementary Information

## Figures and Tables

**Table 1:** Baseline characteristics of the study population.

Characteristic	Patients (n = 130)	Controls (n = 130)	*p*-value
Age (years)	71.2 ± 9.0	71.1 ± 8.7	0.983
Male (%)	79 (60.8)	79 (60.8)	1.000
SBP (mmHg)	152.1 ± 22.3	140.2 ± 19.8	< 0.001
DBP (mmHg)	79.7 ± 12.3	79.5 ± 10.5	0.914
BMI (kg/m^2^)	22.60 ± 3.04	23.2 ± 3.27	0.104
FBG (mmol/L)	5.37 (4.82, 5.85)	5.01 (4.66, 5.56)	0.007
TG (mmol/L)	1.31 (1.02, 1.72)	1.45 (1.05, 1.99)	0.095
TC (mmol/L)	4.62 ± 0.96	4.50 ± 1.03	0.348
HDL-C (mmol/L)	1.12 (1.01, 1.36)	1.19 (1.01, 1.40)	0.466
LDL-C (mmol/L)	2.50 (2.16, 3.20)	2.41 (1.90, 3.14)	0.055
HbA_1C_ (%)	5.8 (5.5, 6.2)	5.6 (5.4, 6.2)	0.116
Homocysteine (μmol/L)	13.1 (10.9, 15.9)	12.4 (9.3, 15.2)	0.294
Serum creatinine (μmol/L)	73.6 ± 17.2	69.7 ± 15.1	0.051
Hs-CRP (mg/L)	3.05 (2.18, 5.8)	2.60 (1.70, 3.60)	<0.001
Hypertension	101 (77.7)	92 (70.8)	0.202
Diabetes mellitus	31 (23.8)	22 (16.9)	0.166
Dyslipidaemia	54 (41.5)	66 (50.8)	0.135
Smokers	52 (40.0)	34 (26.2)	0.018
Alcohol consumers	26 (20.0)	19 (11.6)	0.251
Hypertension med use	33 (32.7^a^)	42 (45.7^a^)	0.065
Diabetes med use	19 (61.3^b^)	16 (72.7^b^)	0.386
NIHSS on day 1	4 (2, 8)	/	/
mRS scores	3 (2, 4)	/	/

The continuous variables are expressed as the mean ± standard deviation (SD) or the median (interquartile range). The categorical values are presented as the frequencies (percentages).

Abbreviations: LAA stroke, large artery atherosclerotic stroke; SBP, systolic blood pressure; DBP, diastolic blood pressure; BMI, body mass index; FBG, fasting blood glucose; TG, triglycerides; TC, total cholesterol; HDL-C, high-density lipoprotein cholesterol; LDL-C, low-density lipoprotein cholesterol; HbA_1C_, haemoglobin A1c; hs-CRP, high-sensitivity C-reactive protein; NIHSS, National Institutes of Health Stroke Scale; mRS, modified Rankin Scale.

^a^Represents the percentage in the hypertension population.

^b^Represents the percentage in the diabetes population.

**Table 2:** Galectin-1, -3, -9, and -3BP levels in the controls and patients and their ORs for ischaemic stroke.

	Controls (ng/ml)	Patients (ng/ml)	*p*-value	Adjusted OR (95% CI)	Adjusted *p*-value^b^
Galectin-1	20.93 ± 5.25	20.37 ± 4.65	0.364	0.986 (0.762, 1.277)	0.915
Galectin-3	7.29 ± 1.60	7.92 ± 1.66	0.002	1.361 (1.055, 1.754)	0.018
Galectin-9	6.49 ± 1.40^a^	7.23 ± 1.31^a^	0.005	1.322 (1.024, 1.705)	0.032
Galectin-3BP	6294 (5019, 7346)	6675 (5468, 8527)	0.011	1.256 (0.981, 1.608)	0.071

Abbreviations: Galectin-3BP, galectin-3 binding protein; OR, odds ratio; CI, confidence interval.

^a^Geometric means ± approximate SD.

^b^Galectin and Galectin-3 BP levels are divided into four layers according to their concentrations, with the lowest layer serving as the reference. The results are adjusted by age, sex, body mass index, hypertension, diabetes, dyslipidaemia, smoking, drinking, homocysteine, creatinine, and hs-CRP.

**Table 3:** Comparison of the functional outcomes in patients.

Characteristic	Favourable outcome	Poor outcome	*p*-value
Age (years)	69.0 ± 9.9	72.5 ± 7.8	0.038
Male (%)	33 (58.9)	37 (60.7)	0.849
BMI (kg/m^2^)	22.60 ± 3.13	22.60 ± 2.98	0.998
Homocysteine (μmol/L)	12.9 (11.2, 15.2)	11.8 (10.6, 15.4)	0.488
Serum creatinine (μmol/L)	72.2 ± 16.8	73.0 ± 17.5	0.797
Hs-CRP (mg/L)	2.55 (1.98, 5.65)	3.95 (2.05, 5.98)	0.084
Hypertension	43 (76.8)	49 (80.3)	0.641
Diabetes mellitus	14 (25.0)	14 (23.0)	0.795
Dyslipidaemia	19 (33.9)	26 (42.6)	0.334
Smokers	20 (35.7)	36 (50.7)	0.091
Alcohol consumers	10 (17.9)	13 (21.3)	0.639
NIHSS on day 1	3 (2, 4)	8 (5, 12)	<0.001
Gal-1 on day 1 (ng/ml)	19.29 (16.79, 21.27)	20.25 (17.83, 23.24)	0.189
Gal-3 on day 1 (ng/ml)	7.48 ± 1.64	8.25 ± 1.66	0.013
Gal-9 on day 1 (ng/ml)	7.21 ± 1.33^a^	7.21 ± 1.29^a^	0.998
Gal-3BP on day 1 (ng/ml)	6642 (5627, 7552)	7020 (5552, 8977)	0.377
NIHSS on day 6	2 (1, 2)	6 (4, 12)	<0.001
Gal-1 on day 6 (ng/ml)	19.53 (16.70, 21.52)	19.59 (17.11, 22.98)	0.688
Gal-3 on day 6 (ng/ml)	7.64 ± 1.80	8.52 ± 2.03	0.024
Gal-9 on day 6 (ng/ml)	7.22 ± 1.42^a^	7.41 ± 1.32^a^	0.679
Gal-3BP on day 6 (ng/ml)	6772 (5884, 7915)	6874 (5803, 8965)	0.591

The continuous variables are expressed as the mean ± standard deviation (SD) or the median (interquartile range). The categorical values are shown as the frequencies (percentages).

A favourable outcome is an mRS score of ≤2 at 3 months post-stroke and a poor outcome is an mRS score of >2 at 3 months post-stroke.

Abbreviations: mRS, modified Rankin Scale; NIHSS, NIH Stroke Scale; BMI, body mass index; hs-CRP, high-sensitivity C-reactive protein; Gal-1, Galectin-1; Gal-3, Galectin-3; Gal-9, Galectin-9; Galectin-3BP, Galectin-3 binding protein.

^a^Geometric means ± approximate SD.
