# Supplementary material for: Serum levels of galectin-1, galectin-3, and galectin-9 are associated with large artery atherosclerotic stroke
Source: Sci Rep. 2017 Jan 23;7:40994. doi: 10.1038/srep40994 (PMC5256273; doi:10.1038/srep40994)
Supplement: Supplementary Information [file srep40994-s1.pdf]

Supplementary Information

The title of the manuscript

Serum levels of galectin-1, galectin-3, and galectin-9 are associated with large artery atherosclerotic stroke

Xin-Wei He[1], Wei-Ling Li[1], Cai Li[1], Peng Liu[1], Yu-Guang Shen[1], Min Zhu[2], Xiao-Ping Jin[1*]

[1] Department of Neurology, Taizhou Hospital, Wenzhou Medical University, Linhai, 317000, China

[2] Medical Research Center, Taizhou Hospital, Wenzhou Medical University, Linhai, 317000, China

[*]corresponding author, hhh841062205@aliyun.com

	Galectin-1	Galectin-3	Galectin-9	Galectin-3BP
Galectin-1	/	$r^c = 0.160^a$<br>$p = 0.069$	$r = 0.324^a$<br>$p < 0.001$	$r = -0.035^a$<br>$p = 0.691$
Galectin-3	$r^c = 0.178^b$<br>$p = 0.043$	/	$r = 0.382^a$<br>$p < 0.001$	$r = 0.181$<br>$p = 0.040$
Galectin-9	$r = 0.339^b$<br>$p < 0.001$	$r = 0.215^b$<br>$p = 0.014$	/	$r = 0.134^a$<br>$p = 0.128$
Galectin-3BP	$r = 0.117^b$<br>$p = 0.184$	$r = 0.180^b$<br>$p = 0.041$	$r = 0.044^b$<br>$p = 0.618$	/

Supplemental Table S1. Correlations between serum levels of galectin-1, galectin-3, galectin-3BP, and galectin-9

[a] Correlation coefficient in controls

[b] Correlation coefficient in patients

r^c represents Pearson's correlation coefficient

r represents Spearman's rank correlation coefficient

Abbreviations: Galectin-3BP, galectin-3-binding protein

	Galectin-1		Galectin-3		Galectin-9		Galectin-3BP	
	r	*p*	r	*p*	r	*p*	r	*p*
NIHSS on day 1	0.086	0.333	0.081	0.362	-0.043	0.624	-0.003	0.961
NIHSS on day 6	0.122	0.210	0.054	0.578	0.021	0.828	-0.082	0.404
Stroke volume	0.133	0.153	0.153	0.099	-0.009	0.927	0.062	0.507

Supplementary Table S2. Correlation of serum levels of galectin-1, -3, -9, and -3BP with stroke severity.

r represents Spearman's rank correlation coefficient.

Abbreviations: Galectin-3BP, galectin-3-binding protein; NIHSS, National Institutes of Health Stroke Scale.

Characteristic	Patients have the second blood samples (n = 109)	All stroke patients (n = 130)	*p* value
Age (years)	70.1 ± 9.0	71.2 ± 9.0	0.390
Male (%)	71 (65.1)	79 (60.8)	0.487
SBP (mmHg)	151.8 ± 22.9	152.1 ± 22.3	0.895
DBP (mmHg)	80.9 ± 12.7	79.7 ± 12.3	0.459
BMI (kg/m^2)	22.80 ± 3.20	22.60 ± 3.04	0.619
FBG (mmol/L)	5.37 (4.81, 6.35)	5.37 (4.82, 5.85)	0.972
TG (mmol/L)	1.32 (1.03, 1.76)	1.31 (1.02, 1.72)	0.811
TC (mmol/L)	4.59 ± 1.00	4.62 ± 0.96	0.853
HDL-C (mmol/L)	1.10 (1.00, 1.31)	1.12 (1.01, 1.36)	0.420
LDL-C (mmol/L)	2.50 (2.17, 3.17)	2.50 (2.16, 3.20)	0.925
HbA_{1C} (%)	5.8 (5.4, 6.2)	5.8 (5.5, 6.2)	0.562
Homocysteine (μmol/L)	12.3 (10.6, 15.5)	13.1 (10.9, 15.9)	0.846
Serum creatinine (μmol/L)	72.4 ± 16.5	73.6 ± 17.2	0.583
Hs-CRP (mg/L)	3.30 (2.08, 5.90)	3.05 (2.18, 5.80)	0.982
Hypertension	86 (78.9)	101 (77.7)	0.822
Diabetes mellitus	25 (22.9)	31 (23.8)	0.869
Dyslipidaemia	46 (42.2)	54 (41.5)	0.918
Smokers	48 (44.0)	52 (40.0)	0.529
Alcohol consumers	23 (21.1)	26 (20.0)	0.834
Hypertension med use	28 (32.6 [a])	33 (32.7 [a])	0.987
Diabetes med use	14 (56.0 [b])	19 (61.3 [b])	0.689
NIHSS on day 1	4 (2, 8)	4 (2, 8)	0.986
mRS scores	3 (2, 4)	3 (2, 4)	0.840

Supplementary Table S3. Baseline characteristics of the subgroup of patients have the second blood samples on day 6 after stroke and comparison to the whole LAA stroke patients.

Continuous variables are expressed as the mean ± standard deviation (SD) or the median (interquartile range). Categorical values are presented as frequencies (percentages).

[a] represents the percentage in the hypertension population.

[b] represents the percentage in the diabetes population.

Abbreviations: LAA stroke, large artery atherosclerotic stroke; SBP, systolic blood pressure; DBP, diastolic blood pressure; BMI, body mass index; FBG, fasting blood glucose; TG, triglycerides; TC, total cholesterol; HDL-C, high-density lipoprotein cholesterol; LDL-C, low-density lipoprotein cholesterol; HbA_{1C}, haemoglobin A1c; hs-CRP, high-sensitivity C-reactive protein; NIHSS, National Institutes of

Health Stroke Scale; mRS, modified Rankin Scale.

Characteristic	Patients have the third blood samples (n = 39)	Patients have the second blood samples (n = 109)	*p* value
Age (years)	69.6 ± 10.6	70.1 ± 9.0	0.752
Male (%)	25 (64.1)	71 (65.1)	0.907
SBP (mmHg)	147.7 ± 25.7	151.8 ± 22.9	0.359
DBP (mmHg)	82.2 ± 12.9	80.9 ± 12.7	0.578
BMI (kg/m^2)	23.20 ± 3.46	22.80 ± 3.20	0.476
FBG (mmol/L)	5.47 (5.04, 7.46)	5.37 (4.81, 6.35)	0.431
TG (mmol/L)	1.28 (1.09, 1.72)	1.32 (1.03, 1.76)	0.804
TC (mmol/L)	4.93 ± 0.94	4.59 ± 1.00	0.071
HDL-C (mmol/L)	1.16 (1.04, 1.37)	1.10 (1.00, 1.31)	0.402
LDL-C (mmol/L)	3.08 (2.49, 3.63)	2.50 (2.17, 3.17)	0.007
HbA_{1C} (%)	5.9 (5.5, 6.8)	5.8 (5.4, 6.2)	0.226
Homocysteine (μmol/L)	14.5 (11.6, 17.6)	12.3 (10.6, 15.5)	0.206
Serum creatinine (μmol/L)	70.2 ± 13.7	72.4 ± 16.5	0.461
Hs-CRP (mg/L)	2.8 (2.13, 6.08)	3.30 (2.08, 5.90)	0.601
Hypertension	31 (79.5)	86 (78.9)	0.938
Diabetes mellitus	12 (30.8)	25 (22.9)	0.332
Dyslipidaemia	19 (48.7)	46 (42.2)	0.482
Smokers	13 (33.3)	48 (44.0)	0.244
Alcohol consumers	9 (23.1)	23 (21.1)	0.797
Hypertension med use	11 (35.5 [a])	28 (32.6 [a])	0.767
Diabetes med use	5 (41.7 [b])	14 (56.0 [b])	0.414
NIHSS on day 1	3 (2, 4)	4 (2, 8)	0.004
mRS scores	3 (1, 3)	3 (2, 4)	0.017

Supplementary Table S4. Baseline characteristics of the subgroup of patients have the second blood samples on day 6 after stroke and third blood samples in 4th week after stroke.

Continuous variables are expressed as the mean ± standard deviation (SD) or the median (interquartile range). Categorical values are presented as frequencies (percentages).

[a] represents the percentage in the hypertension population.

[b] represents the percentage in the diabetes population.

Abbreviations: LAA stroke, large artery atherosclerotic stroke; SBP, systolic blood pressure; DBP, diastolic blood pressure; BMI, body mass index; FBG, fasting blood glucose; TG, triglycerides; TC, total

cholesterol; HDL-C, high-density lipoprotein cholesterol; LDL-C, low-density lipoprotein cholesterol; HbA_{1C}, haemoglobin A1c; hs-CRP, high-sensitivity C-reactive protein; NIHSS, National Institutes of Health Stroke Scale; mRS, modified Rankin Scale.
